# Flourishing, psychological distress and internalized stigma among parents of an adult son or daughter with schizophrenia

**DOI:** 10.1177/00207640231166630

**Published:** 2023-04-24

**Authors:** Loukia Dimitriou, Marcus Chiu, Jerome Carson

**Affiliations:** 1 School of Psychology, University of Bolton, Bolton, UK; 2 School of Health and Social Care, University of Bolton, Bolton, UK

**Keywords:** Parents, schizophrenia, internalized stigma, flourishing, psychological distress

## Abstract

**Background:**

Parents of adults diagnosed with schizophrenia, have been reported to have higher levels of psychological distress than the general population, and parents whose offspring have other mental or physical illnesses.

**Aim:**

This study examines the comparatively new construct of flourishing, and its relationship to internalized stigma and psychological distress.

**Method:**

A cross-sectional survey was conducted between July 2021 and March 2022, with an international sample of 200 parents of adult sons or daughters diagnosed with schizophrenia. Participants completed a demographic questionnaire and three standardized inventories. These were the PERMA Profiler, which measures flourishing, the CORE-10, which measures psychological distress, and a new parental Internalized Stigma Scale. Sample characteristics of individuals of schizophrenia and their parents were examined using descriptive statistics, and the contributing factors affecting stigma were assessed through regression analysis.

**Results:**

The initial hypothesis that parents scoring *high* on internalized stigma, would have significantly higher levels of psychological distress and lower levels of flourishing, than parents with *low-*level internalized stigma, was confirmed. Overall, the flourishing levels were lower and psychological distress higher in these parents, than those of the general population. Regression analysis identified psychological distress and hopefulness as the two major predictors of flourishing, though in different directions. Interestingly, stigma did not determine flourishing, in spite of their close relationship.

**Conclusions:**

Researchers have long been aware of internalized stigma in persons with schizophrenia. Yet this study is one of the few that linked it with parents of adults with schizophrenia and flourishing and psychological distress. Implications were discussed in the light of the findings.

## Introduction

### Burden of family caregivers

Being a parent of an adult son or daughter with schizophrenia can have detrimental effects on parental mental and physical health. Given the high unemployment levels in persons with schizophrenia, many of them continue living at home, being cared for by their parents, leading at times to significant levels of caregiver burden (Saunders, 2003). Problems of stress, aggression, chronic sadness, immense stigma, role shifts, social retreat and financial/career issues are just a few of the problems faced by parents, all of which are compounded by a lack of resources and assistance (K. J. Chang et al., 2018; Kageyama et al., 2018; Koschorke et al., 2017; Olwit et al., 2015). The way that schizophrenia progresses, has an impact on parents, where lower levels of functioning in persons with schizophrenia are linked to higher levels of stress, anxiety and depression in the parents (Flyckt et al., 2013; Gater et al., 2014). Research reveals that parents who were caring for a son or daughter with schizophrenia had a significant loss of productivity themselves (Gupta et al., 2015a, 2015b). Surprisingly given the chronic nature of schizophrenia only a small percentage of parents (10%), receive any kind of intervention from mental health services (Cohen et al., 2006), even though recent research has suggested that it could be helpful for the parents in enabling them to better manage their son or daughters’ condition (Forcheron et al., 2023).

Of those parents who sought support for themselves and their son or daughter with schizophrenia, many judged mental health interventions to be ineffective (Ferriter & Huband, 2003). Furthermore, family members and parents mentioned how difficult it was for them to seek assistance and how hard it was for them to receive adequate information about access to mental health treatments, as well as financial problems (Czuchta & McCay, 2001; Jakobsen & Severinsson, 2006; Reid et al., 2005). Furthermore, many parents may experience self-stigma themselves due to their adult son or daughter having schizophrenia, so-called internalized stigma. This can be described as embracing stereotypes about the mental illness, social withdrawal and the perceived discrimination that sufferers receive (C. C. Chang et al., 2017; Frost, 2011; Livingston & Boyd, 2010). A family member (particularly parents) of an individual with a mental health disorder, for example, was subjected to substantially greater levels of stigma than their peers, who did not have an individual with a mental health problem within their family (Kassam et al., 2012; Krupchanka et al., 2018). Furthermore, research indicated that family guilt was much more common in families of those with mental illness, than in families of people with cancer (Ohaeri & Fido, 2001), reflecting the inequality of perception between mental health and physical health.

### Flourishing

It was Martin Seligman who argued that increasing flourishing was to be the goal of Positive Psychology (Seligman, 2011). Seligman argued that flourishing comprised *p*ositive emotions, *e*ngagement, *r*elationships, *m*eaning and *a*ccomplishments (PERMA). In 2015, Kern et al. (2015) provided the field with the most reliable and valid measure of flourishing, the PERMA Profiler. The PERMA Profiler Scale may provide an additional outcome measure for researchers delivering interventions with parents, as well as enabling comparison with normative population samples. While Keyes (2002) was the first person to establish a link between flourishing-languishing and mental health, the concept of flourishing has thus far not been studied in parents who have adult sons or daughters with schizophrenia.

The present study sought to examine flourishing levels, psychological distress and internalized stigma in an international group of parents, with adult sons and daughters who were diagnosed with schizophrenia. Apart from the initial prediction that parents who scored High on internalized stigma would score significantly lower on flourishing and higher on psychological distress than parents Low in stigma, this study did not hold out any other presumptions because of the lack of research on flourishing in these parents.

## Methods

### Procedure and ethics

This online cross-sectional study was conducted between July 2021 and March 2022. The target population was parents who had an adult son or daughter with schizophrenia. Participants were adults, age range 38 to 81, mean = 56.41, standard deviation = 9.97. Participants were recruited using opportunity sampling, largely via targeted posts on various Facebook support groups and pages with people with schizophrenia and their relatives. Additional sources of participants were national organizations and communities of parents with schizophrenia such as EUFAMI, the European Federation of Associations of Families of People with Mental Illness and SABDA, the Schizophrenia and Bipolar Disorder Alliance. The study was given ethical approval by the Psychology Department at the University of XXXX, in line with British Psychological Society Guidelines (British Psychology Society, 2021). Participation in the survey was voluntary and without any monetary or material incentives.

### Measures

The demographic questionnaire comprised 28 questions, 6 about the parents, including age, gender, number of children, country of residence and if they were a primary carer and had a disability themselves. There were eight questions about their adult son or daughter including their age, when they were diagnosed, their age now, if they lived with them, if they received welfare benefits, medication, hospitalization and employment status. There were three questions on a 10-point scale, which asked the participant about access to mental health services and helpfulness, as well as how hopeful they felt about their son or daughter’s future? Additionally, there were five Covid-19 related questions, about the impact on the participants and their son or daughter, three closed type questions (Yes/No) and two open questions. A further six questions asked about place of residence, specific difficulties they faced with mental health, the information they had about schizophrenia and the negatives and the positives in their life due to the schizophrenia diagnosis of their son or daughter. The survey used three standardized self-report questionnaires: The PERMA profiler; CORE-10 and PISMI (Parents Internalized Stigma of Mental Illness Inventory). At the end an optional section asked for the personal e-mail of the participants if they wanted to learn the outcome of the survey. (Copies of the measures used and the SPSS datafile are available from the authors).

1. PERMA. This is a 23-item measure of flourishing developed by Kern et al. (2015; Butler & Kern, 2016). Some 15 items measure PERMA itself (Positive emotions, Engagement, Relationships, Meaning and Accomplishments). There are additional three item subscales measuring Physical Health and Negative Emotions, along with a Loneliness item and an Overall Happiness item. The flourishing score is derived from adding the PERMA Total to the Overall Happiness item. This scale has been extensively validated, both by the developers and independent researchers (Ascenso et al., 2018; Ryan et al., 2019). All items are scored on a 0 to 10 scale. The internal consistency is high, ranging from .92 to .95, with test-retest reliability ranging from .69 to .88. The internal reliability of the PERMA Scale in this study (15 item score) was .94.
2. Clinical Outcomes in Routine Evaluation (CORE-10). This is a shorter 10-item scale which has been extracted from the larger CORE-OM (Connell & Barkham, 2007). This scale has also been extensively validated (Barkham et al., 2013). It is a measure of psychological distress. Items are rated on a five-point frequency of occurrence basis. For instance, ‘I have felt tense or anxious’. This is rated as, ‘Not at all’, ‘Only occasionally’, ‘Sometimes’, ‘Often’ or ‘Most or all of the time’. Internal consistency is high at .90, with a sample size of more than 5,000 participants. The internal reliability of the CORE-10 in this study was .86.
3. Finally, The Parents’ Internalized Stigma of Mental Illness (PISMI) Scale has 12-items that break down into three subscales, discrimination experience, social withdrawal and alienation, and stereotype endorsement. It uses a 4-point Likert scale with answers ranging from ‘strongly disagree’ to ‘strongly agree’. Internal consistency of the 12-item PISMI gives a reliability coefficient α of .76 with Cronbach’s α of .78 for discrimination experience, .65 for social withdrawal and alienation, and .61 for stereotype endorsement. The internal reliability of the 12-item PISMI in this study was .87, with Cronbach’s α being .80 for discrimination experience, .81 for social withdrawal and alienation and .75 for stereotype endorsement.

### Statistical analysis

Data analysis was conducted using the statistical software package SPSS (version 27). The data from Google Forms were imported via MS Excel, checked, cleaned and then imported into SPSS. There were no incomplete survey results, as the use of mandatory field completion had been incorporated into the Google Forms design, except for the age category. Any missing values, for example where a reply was not applicable, were coded as ‘999’. Before statistical analysis was undertaken, testing for normality was carried out on the three dependent variables. On the Kolmogorov-Smirnov Tests, all three dependent variables were found to be significantly different from the normal distribution. Skewness rates and visual inspection of data bar charts confirmed this. The dataset was not affected by outliers, as the means and the 5% trimmed means were similar. Therefore, the data were analysed using non-parametric statistics.

## Results

### Descriptive statistics of the sample population

Demographic characteristics of the participants are summarized in Table 1. The sample consisted of 200 participants (182 mothers and 18 fathers). As expected, women formed the largest group of respondents as they are often the primary caregivers. More than half of the participants were from North America. Only 32% of the ‘ill persons’ were in employment of any kind and a surprising 88.5% were single. These are well below community employment rates and the number of adults in relationships, showing the devastating psychosocial effects of schizophrenia.

**Table 1.** Demographic features of the participants (*N* = 200).

	*N* (%)
Gender	
Male	18 (9.0)
Female	182 (91.0)
Age of parents	
38–56 years old	96 (48.0)
57–81 years old	100 (50.0)
Missing data	4 (2.0)
Country/location	
USA and Canada	125 (62.5)
UK, England and Scotland	28 (14.0)
Europe	12 (6.0)
Australia and New Zealand	19 (8.5)
Africa	10 (5.0)
Asia	7 (3.5)
Number of children	
1	30 (15.0)
2	79 (39.5)
3	60 (30.0)
4	20 (10.0)
5 and more	11 (5.5)
Disability of parents	
Yes	32 (16.0)
No	168 (84.0)
Primary carer	
Yes	158 (79.0)
No	42 (21.0)
Age of the first diagnosis	
0–20	95 (47.5)
21–28	105 (52.5)
Age of adult children now	
18–27	87 (43.5)
28–53	106 (53.0)
Missing data	7 (3.5)
Adult children with a paid job	
Yes	32 (16.0)
No	168 (84.0)
Personal status of adult children	
Single	177 (88.5)
In a relationship	12 (6.0)
Divorced	7 (3.5)
Married	3 (1.5)
Widow/er	1 (0.5)
Receive medication	
Yes	163 (81.5)
No	37 (18.5)
Adult child psychiatric hospitalization: Y/N	
Yes	179 (89.5)
No	21 (10.5)
Children receive welfare benefits from government	
Yes	128 (64.0)
No	72 (36.0)

Descriptive statistics for the sample are provided in Table 2. There are reliable comparative data on the PERMA Scale and also CORE-10 from a very large community survey, *n* = 1,608, conducted at the start of the Covid-19 pandemic (Carson et al., 2020). The mean score from that study on PERMA Flourishing was 101.47 (*SD* = 27.73). This contrasts with a Flourishing score of 92.15 (*SD* = 30.43) from the current sample. Using the ‘Summary Independent Samples *t* test’ function in SPSS, this gives a value of: *t* = −4.423, *df* = 1,805, *p* = .001, effect size = .33 (Hedge’s ‘g’). Parents in the current study had significantly lower levels of flourishing than this community sample. On the CORE-10, the mean score from the Carson et al. (2020) study was 12.82 (*SD* = 7.61), compared to 16.63 (*SD* = 7.81) in the current study: *t* = 6.643, *df* = 1,805, *p* = .001, effect size = .50. This shows significantly higher levels of psychological distress in the current sample.

**Table 2.** Means, standard deviations and range for study dependent variables.

Scales (*N* = 200)	Min	Max	Mean	*SD*
PISMI discrimination experience	4	16	7.61	3.15
Social withdrawal/alienation	4	16	8.19	3.57
Stereotype endorsement	4	16	7.50	2.99
PISMI total	12	45	23.29	7.94
PERMA positive emotions	0	28	15.80	6.78
Engagement	0	28	17.18	5.93
Relationships	1	30	17.64	7.06
Meaning	0	30	18.10	7.10
Personal accomplishments	2	29	17.88	5.56
PERMA total	15	139	86.58	28.14
Physical health	0	30	16.77	7.12
Negative emotions	3	30	17.46	6.18
Total score on flourishing	15	149	92.08	30.37
Total score on CORE-10	2	38	16.64	7.79

The situation is more complex with respect to the Parental Internalized Stigma of Mental Illness Scale. The authors are only aware of one study by Zisman-Ilani et al. (2013), which has presented data on this scale. Their study had a sample of 180 parents with adult children with serious mental illness, individuals with a diagnosis of schizophrenia comprised 53.1%, whereas this was 100% in our sample. Similar numbers lived with their parents, 65.9% compared to 60% in our study, with a higher percentage of fathers participating in their study 16.5 and 9% in our survey respectively (Zisman-Ilani et al., 2013). The Parental Internalized Stigma questionnaire has 12 items divided into three subcategories. The first category was *discrimination experience*. The two samples were not significantly different (Israeli sample mean = 1.85, *SD* = 0.62, Present study mean = 1.90, *SD* = 0.79). On *social withdrawal and alienation*, our sample was compared to the Israeli sample (Israeli sample mean = 2.23, *SD* = 0.63, Present study mean = 2.05, *SD* = 0.89), which means our sample experienced lower social withdrawal and alienation. Moreover, in the last subcategory, the present research sample experienced a lower *stereotype endorsement* compared to the Israeli sample (Israeli sample mean = 2.30, *SD* = 0.59, Present study mean = 1.87, *SD* = 0.75). When the comparison is between the *total Stigma Scale* scores our sample experienced lower stigma than the Israeli sample (Israeli sample mean = 2.13, *SD* = 0.46, Present study mean = 1.94, *SD* = 0.66).

In this the Parental Internalized Stigma Scale (PISMI), participants scored more highly (⩾2.0). This was on 7 items (see Table 3).

**Table 3.** Mean and standard deviation of the Parental Internalized Stigma Scale (PISMI).

	Mean	*SD*
7. I don’t talk about my son or daughter much because I don’t want to burden others with his/her mental illness.	2.36	1.10
10. People having a son or daughter with mental illness cannot live a good, rewarding life.	2.13	0.99
11. Having a son or daughter with mental illness has spoiled my life.	2.11	1.12
8. I feel inferior to others who don’t have a son or daughter with mental illness.	2.09	1.20
6. I avoid telling people that I have a son or daughter with mental illness.	2.08	1.09
4. Stereotypes about the mentally ill also apply to me.	2.02	1.02
2. Nobody would be interested in getting close to me because I have a son or daughter with mental illness.	1.99	1.07

### Inferential statistics

Hypothesis: Parents categorized as HIGH on Internalized Stigma will score significantly lower on flourishing and higher on psychological distress than parents scoring LOW on Internalized Stigma.

Participants scoring from 12 to 21.5 were categorized as LOW Internalized Stigma (*n* = 96), and those scoring between 21.6 to 45 (*n* = 104) were categorized as HIGH Internalized Stigma. This was done on the basis of a median split.

The hypothesis is supported by the results. Parents scoring HIGH on internalized stigma, had significantly higher levels of psychological distress and lower levels of flourishing, than parents with LOW levels of internalized stigma. On the other hand, those parents who were categorized as being in the LOW-stigma group, enjoyed much higher scores on every domain of positive emotions (Engagement, Relationships, Meaning and Accomplishment), and better physical health when compared to the HIGH-stigma group (see Table 4).

**Table 4.** Mean scores on PERMA scale for parents with high internalized stigma versus those with low internalized stigma.

	High internalized stigma (*n* = 96)	Low internalized stigma (*n* = 104)	*z* Score *p* value	Effect size
	Mean (*SD*)	Mean (*SD*)		
PERMA positive emotions	13.79 (6.50)	17.71 (6.53)	*z* = −4.158 *p* = .001	0.29
Engagement	15.72 (5.43)	18.54 (6.10)	*z* = −3.782 *p* = .001	0.27
Relationships	15.71 (6.16)	19.43 (7.43)	*z* = −4.042 *p* = .001	0.28
Meaning	15.76 (6.16)	20.30 (6.89)	*z* = −4.683 *p* = .001	0.33
Accomplishment	16.36 (5.09)	19.32 (5.64)	*z* = −3.983 *p* = .001	0.28
PERMA total	77.34 (24.43)	95.30 (28.83)	*z* = −5.022 *p* = .001	0.36
Negative emotions	19.36 (5.92)	15.65 (5.92)	*z* = −3.933 *p* = .001	0.28
Physical health	15.52 (6.96)	17.94 (7.13)	*z* = −2.646 *p* = .001	0.19
Total flourishing	82.07 (26.52)	101.53 (30.95)	*z* = −4.980 *p* = .001	0.35

Table 5 below, showed clearly the HIGH-stigma group has significantly higher symptom scores on CORE-10 (Mean = 19.80; *SD* = 7.71), than the LOW stigma group (Mean = 13.68; *SD* = 6.70). A closer examination revealed that all individual item scores differed significantly between the two groups except 1 item. The difference between the two groups (High: Mean = 2.21, *SD* = 1.11 vs. Low: Mean = 1.83, *SD* = 1.35) did not reach statistical significance on ‘Someone to turn to for support’. It was likely caused by the greater variation within the LOW-stigma group.

**Table 5.** Mean scores on CORE-10 scale by HIGH/LOW internalized stigma group.

Item	High internalized stigma (*n* = 96)	Low internalized stigma (*n* = 104)	*z* Score *p* value	Effect size
Tense, anxious or nervous.	2.85 (1.03)	2.22 (1.07)	*z* = −4.035 *p* = .001	0.28
Someone to turn to for support.	2.21 (1.11)	1.83 (1.35)	*z* = −1.900 *p* = .057	0.13
Able to cope when things go wrong.	1.86 (1.00)	1.31 (1.02)	z=−3.793 *p* = .001	0.27
Talking to people is too much.	2.19 (1.08)	1.45 (1.07)	*z* = −4.620 *p* = .001	0.33
I have felt panic or terror.	1.66 (1.28)	0.92 (1.06)	*z* = −4.131 *p* = .001	0.29
I made plans to end my life.	0.46 (0.81)	0.19 (.58)	*z* = −2.899 *p* = .001	0.20
Difficulty getting to sleep or staying asleep.	2.33 (1.37)	1.94 (1.29)	*z* = −2.121 *p* = .004	0.15
I have felt despairing or hopeless.	2.09 (1.27)	1.26 (1.13)	*z* = −4.576 *p* = .001	0.32
I have felt unhappy.	2.44 (1.13)	1.61 (1.04)	*z* = −5.069 *p* = .001	0.36
Unwanted images or memories have been distressing me.	1.71 (1.38)	0.93 (1.04)	*z* = −4.048 *p* = .001	0.29
Total score on CORE-10	19.80 (7.71)	13.68 (6.70)	*z* = −5.610 *p* = .001	0.40

To control for multiple comparisons a Bonferonni adjustment was calculated. Hence, .05 ÷ 11 = .0045.

Only values lower than this are counted as significant. Only one of the above comparisons, *‘I have felt I have someone to turn to for support when needed’, failed to reach significance. On every other* comparison the HIGH Stigma group scored worse than the LOW Stigma group.

Correlational analysis using Spearman’s Rho, showed that CORE-10 correlated highly but negatively with Flourishing, *r* = −.673 and similarly with Stigma, *r* = −.476. It was evident that higher levels of psychological distress and internalized stigma are associated with lower levels of flourishing. On the other hand, CORE-10 correlated positively and significantly with stigma (*r* = .526).

Multiple regression was conducted to see which variables best predicted total Flourishing? Five variables accounted for 49.1% of the variance. The most salient predictor was psychological distress (β = −.579), but negatively, meaning the higher the parents scored on psychological distress the more likely they were to have a lower Flourishing score. The next best predictor of Flourishing was hopefulness about the future (β = .167). This demonstrated that the more hopeful for the future the parent was, the more likely they were to score higher in flourishing. Age, stigma and access to services were not significant predictors of flourishing levels (Table 6).

**Table 6.** Model summary of the regression analysis.

Model	*R*	*R* square	Adjusted *R* square	*SE* of the estimate
5.	.710^e^	.504	.491	21.66

e Predictors: (Constant), Total score on CORE 10, Age, Stigma total score, Do you feel hopeful for the future of your son or daughter? Have you found it easy to access to mental health services for your son/daughter?

f Dependent Variable: Flourishing Total Revised The model shows that the five variables accounted for 49.1% of the total variance in the Flourishing score.

**Table:** *Coefficients*.

Model	Unstandardized *B*	Coefficients *SE*	Standardized coefficients β	*t*	Sig.	Collinearity tolerance	Statistics VIF
5							
(Constant)	122.626	12.241		10.018	.000		
Total score on CORE 10	−2.255	0.217	−.579	−9.358	.000	0.681	1.468
Age	0.080	0.183	.023	0.436	.663	0.930	1.075
Stigma total score	−0.346	0.231	−.090	−1.496	.136	0.713	1.403
Do you feel hopeful for the future of your son or daughter?	1.924	0.642	.167	3.000	.003	0.841	1.189
Have you found it easy to access to mental health services for your son/daughter?	0.722	0.628	.066	1.150	.251	0.802	1.247

## Discussion

From the demographic questionnaire, it is evident that most ill sons or daughters were both unemployed and single. This shows the pervasive nature of the impact of the schizophrenia on the person’s work competence, and capacity for confiding relationships. It will not do them justice if support services for capacity building or personal recovery do not target enhancing one’s employability and capacity to initiate and sustain confiding relationships. The profile of primary caregivers was also striking. They were predominantly mothers/women (91%); half of them were over 60 and one-sixth reported having a disability. Family caregiving could have become harder when limiting factors are added together (losing spouse, being aged and disabled). It points to the urgent need for comprehensive planning rather than ad hoc or ‘wait-and-see’ strategies for primary caregivers. Gender issues in family caregiving of adult children with schizophrenia did not emerge as significant in terms of psychological distress, stigma and flourishing. Previous research on gender effects in caregivers are equivocal, with some studies suggesting that both genders were equally vulnerable to caregiving stressors (Ghosh & Greenberg, 2012), but mothers are usually the primary caregivers of individuals with schizophrenia. If the latter is the case, it may end up that more mothers reported having experienced higher levels of psychological distress (Reinhard et al., 2008). It is inconclusive in this study since there were too few cases of male carers to make a meaningful comparison.

Another finding that drew attention was that parents, who had HIGH-levels of internalized stigma, experienced significantly higher level of psychological distress, but significantly lower levels of Flourishing, than those parents with LOW-levels of stigma. Self-perceived stigma has been associated with creating a sense of inferiority, poor self-image, reduced motivation to seek help and creating psychological dissonance (Chiu et al., 2013, 2015; Lee et al., 2005). As a result, it is probable that internalized stigma will cause greater psychological distress. What this study added is that they were also the ones who had a lower capacity for flourishing. Although the relationship between the two (i.e. stigma and flourishing) was expected to be in the opposite direction, the pathway remained unclear, and for the first time, the impact appeared to have general adverse impacts on all dimensions of flourishing. Not only did it keep people disengaged and isolated, it also diminished one’s capacity for relationship building and positive meaning making, and deprived people of their sense of personal accomplishments. Previous studies with parents of individuals with schizophrenia have shown more social isolation and a lower quality of life compared to the general population (Hayes et al., 2015). Other studies have found that carers of people with schizophrenia had significant social isolation or a negative impact in their social life and relationships (Jagannathan et al., 2014; Suro & Weisman de Mamani, 2013; Zhou et al., 2016). In this study, 71% of the participants felt isolated because of their son or daughter’s diagnosis. Compared to those who did not feel isolated, they had significantly lower flourishing levels, higher psychological distress and higher internalized stigma as well as having more difficulty accessing mental health services and felt significantly less hopeful about their future. It told of the daunting tasks of caring for people with schizophrenia in the family, and the absence/presence of social isolation may be a reliable indicator of needs for services to look out for.

Regression analysis identified psychological distress and hopefulness as the two major predictors of the level of flourishing, though in different directions. High levels of psychological distress disrupted peace of mind, shaped negative schema and therefore limited one’s ability to develop positive aspects of flourishing. It is therefore suggestive that the psychological distress of the family carer should be the prime intervention target before any other/further objectives. Other interventions will become more effective when psychological distress is brought down to a reasonable level. On the other hand, clinical or social interventions will need to generate a sense of hopefulness. Practitioners may like to adopt a dual approach of reducing distress and building hope. The divided strategy of intervention on either aspect alone may not be as helpful. There are also implications from the result that stigma did not determine flourishing, in spite of their close relationship. As stigma level is not a determinant for flourishing, stigma may not be a concern for families who did not feel particularly distressed and anti-stigma should be part of a comprehensive intervention rather than a stand-alone programme. Up to this moment, the findings appeared to have suggested more holistic programme planning is needed for these family caregivers. Having said that, it has previously been noted that internalized stigma is commonly found among parents (Zisman-Ilani et al., 2013), and has to be worked on, in addition to problem solving and communication skills training (Kuipers et al., 2002; Leff, 2005).

### Limitations

Self-referred research participants and the use of self-report measures, with a cross-sectional data collection method are prone to limitations and biases. The parents made the decision that their adult sons or daughters had a diagnosis of schizophrenia, not the researchers. There is always the possibility of parents reporting the ‘worse-case scenario’ among those who took part in the study. The use of the English language may have limited the scope of the study, and may reduce the nuances of different cultures. Therefore, the results must be interpreted with caution, and future longitudinal studies are needed. Yet on the other hand, an independent study like this has avoided the problem of social desirability that may commonly exist in existing service parent relationships. Anonymity, the format of the structured questionnaire survey, and the international composition of the sample, may provide more comfortable psychological distance, rather than being so closely watched and examined. In-depth interviews and other qualitative methods will be helpful in elucidating the detailed dynamics behind parental internalized stigma (Braun & Clarke, 2022).

## Conclusions

Severe mental illnesses (SMI) like schizophrenia not only affect the sufferer but their family also, especially family carers, who are also more adversely affected by ‘courtesy stigma’. On the practical side, family carers, especially parents represent family resources available to cope with the situations associated with SMI. The poor mental health of family carers, is a good enough reason to justify intervention. The issue of stigma may have to be tackled through a concerted effort at different levels. Certainly, a sense of hope and flourishing has to be restored among family carers before they can fully support the person in recovery. While traditionally researchers have focused on anxiety, depression and carer burden, the present study moves the focus onto flourishing. How can we help carers flourish and get the most out of life? It is important that researchers continue their efforts to further understand the impact of internalized stigma in parents with sons and daughters diagnosed with schizophrenia, because this a relatively new area in mental health care that has not been adequately researched. Brief psychoeducation sessions to parents about internalized stigma, may help reduce internalized stigma. Despite the huge advances in psychopharmacology, cognitive behavioural approaches and family interventions, much work remains to be done, not just for the persons with schizophrenia who experience this devastating mental illness, but also for their parents.
